# Inhalant Mammal-Derived Lipocalin Allergens and the Innate Immunity

**DOI:** 10.3389/falgy.2021.824736

**Published:** 2022-01-27

**Authors:** Tuomas Virtanen

**Affiliations:** Department of Clinical Microbiology, Institute of Clinical Medicine, University of Eastern Finland, Kuopio, Finland

**Keywords:** allergens, animal allergens, lipocalin allergens, lipocalins, allergenicity, allergic sensitization, innate immunity, adaptive immunity

## Abstract

A major part of important mammalian respiratory allergens belongs to the lipocalin family of proteins. By this time, 19 respiratory mammalian lipocalin allergens have been registered in the WHO/IUIS Allergen Nomenclature Database. Originally, lipocalins, small extracellular proteins (molecular mass ca. 20 kDa), were characterized as transport proteins but they are currently known to exert a variety of biological functions. The three-dimensional structure of lipocalins is well-preserved, and lipocalin allergens can exhibit high amino acid identities, in several cases more than 50%. Lipocalins contain an internal ligand-binding site where they can harbor small principally hydrophobic molecules. Another characteristic feature is their capacity to bind to specific cell-surface receptors. In all, the physicochemical properties of lipocalin allergens do not offer any straightforward explanations for their allergenicity. Allergic sensitization begins at epithelial barriers where diverse insults through pattern recognition receptors awaken innate immunity. This front-line response is manifested by epithelial barrier-associated cytokines which together with other components of immunity can initiate the sensitization process. In the following, the crucial factor in allergic sensitization is interleukin (IL)-4 which is needed for stabilizing and promoting the type 2 immune response. The source for IL-4 has been searched widely. Candidates for it may be non-professional antigen-presenting cells, such as basophils or mast cells, as well as CD4+ T cells. The synthesis of IL-4 by CD4+ T cells requires T cell receptor engagement, i.e., the recognition of allergen peptides, which also provides the specificity for sensitization. Lipocalin and innate immunity-associated cell-surface receptors are implicated in facilitating the access of lipocalin allergens into the immune system. However, the significance of this for allergic sensitization is unclear, as the recognition by these receptors has been found to produce conflicting results. As to potential adjuvants associated with mammalian lipocalin allergens, the hydrophobic ligands transported by lipocalins have not been reported to enhance sensitization while it is justified to suppose that lipopolysaccharide plays a role in it. Taken together, type 2 immunity to lipocalin allergens appears to be a harmful immune response resulting from a combination of signals involving both the innate and adaptive immunities.

## Introduction

Allergic diseases constitute a serious health problem in westernized countries. Their development appears to be associated with modern life style, such as the use of antibiotics, high-level hygiene, the lack of exposure to farming-related microbes, or certain infections (1, 2). A high proportion of Europeans (30–50%) showed a positive skin prick test result to at least one of the allergens examined, which is a finding indicating a widespread predisposition for allergy (3). This predisposition, atopy, is a genetic property of an individual to mount an IgE response to innocuous environmental substances, allergens, and thereby become sensitized to them.

The characteristic feature of IgE-mediated allergy is the immediate allergen-induced reaction in which mast cells and basophil granulocytes release a variety of immune mediators in a few minutes (4). In inhalant allergy, the symptoms derive from eyes, airways or systematically. Respectively, they manifest as allergic rhinoconjunctivitis, allergic asthma or anaphylaxis. In the long run, persistent allergic inflammation can lead to irreversible consequences, such as airway tissue remodeling in asthma (5). Before these events can take place, an individual has to become sensitized to allergenic material. It comprises the development of type 2 immune response by CD4+ T cells that enables the synthesis of specific IgE by B cells.

Furry animals are significant sources of allergens in various indoor environments. Exposure to these allergens is usually perennial. As owning pets is very common, exposure to pet allergens does not require a direct contact with pets; the allergens are widely present in homes without pets, schools, day care centers and other environments, at levels able to sensitize, and cause symptoms (6, 7). It is estimated, for example, that about half of the households in Great Britain have either at least one cat or dog (8). Sensitization to horse allergens is common because of riding as a hobby, or occupation (9). Other common sources of pet allergens are mice, rats, guinea pigs, gerbils, and rabbits. Sensitization to rodents can also result from housing conditions or occupational exposure (10–12).

Mammalian inhalant allergens are especially associated with severe allergic disorders, including asthma (7, 13). They are mainly found in a few protein families which are lipocalins, serum albumins, and secretoglobins (14). Almost all important mammalian respiratory allergens belong to the lipocalin family of proteins, the major exception being the cat secretoglobin, Fel d 1 (14). Currently, Allergen Nomenclature Database by the World Health Organization/International Union of Immunological Societies (WHO/IUIS) Allergen Nomenclature Sub-Committee (http://www.allergen.org) lists 19 inhalant mammalian lipocalin allergens. Sensitization to three or more mammalian lipocalin allergens is associated with severe asthma in children (15). In dog allergy, sensitization to lipocalin allergens is characteristic (16).

The purpose of this review is to assess the major components of innate immunity in defining and shaping the allergenic characteristics and features of mammalian lipocalin allergens. Although innate immunity offers some cues for the allergenic capacity of lipocalins, it largely remains enigmatic.

## Lipocalins

Lipocalin proteins belong to the calycin superfamily together with fatty acid-binding proteins, avidins, triabin, and a group of metalloproteinase inhibitors (17, 18). They form a large protein family (https://prosite.expasy.org/PS00213) comprising proteins from vertebrate and invertebrate animals, plants, and bacteria. Sequence homology, biological function, and structural similarity need to be in agreement for a protein to be included in the family (https://prosite.expasy.org/PDOC00187). Lipocalins are typically small extracellular molecules [about 20 kDa without posttranslational modifications (17)] with a variaty of biological functions. Originally, they were described as transport proteins. Mammalian lipocalins are mostly produced in the liver or secretory glands (19). There are 19 lipocalin genes encoding functional proteins in the human genome (18).

The length of lipocalin sequences varies between 160 and 230 amino acids (17) and is around 160 amino acids with lipocalin allergens (Table 1). The sequencial identity between lipocalin proteins is low, in general at the level of 20–30%. Most lipocalins contain one or more intramolecular disulfide bonds stabilizing the structure. Lipocalins can be N- and/or O-glycosylated or non-glycosylated (Table 1).

**Table 1:** Physicochemical characteristics and sensitization rates of mammal-derived inhalant lipocalin allergens^a^.

**Allergen**	**Animal**	**Sensitization^**b**^, %**	**MM^**c**^, kDa**	**Amino acids**	**Isoelectric point**	**Glycosylation**	**Oligomeric state^**d**^**	**UniProtKB accession #^**e**^**	**Structure, PDB entry^**f**^**	**Key reference(s)**
Bos d 2	Cow	90	20	156	4.2	No	M/D	Q28133	1BJ7	(20)
Can f 1	Dog	48	22–25	156	5.2	Yes	D	O18873		(21)
Can f 2	Dog	23	22–27	162	4.9	Putative	M/D/T	O18874	3L4R	(21)
Can f 4	Dog	36	16–18	158	6.2	No	M/D	D7PBH4	4ODD	(22)
Can f 6	Dog	50	27, 29	175	4.9	Putative		H2B3G5		(23)
Cav p 1	Guinea pig	*~80*	20	148	4.3	No	M/D	A0A484HM70		(24, 25)
Cav p 2	Guinea pig	*62–65*	17	154	4.3–4.5	No		P83508		(25, 26)
Cav p 3	Guinea pig	*45–54*	18	155	5.2	No		F0UZ12		(25, 26)
Cav p 6	Guinea pig	*59*	18	162				S0BDX9		(25)
Equ c 1	Horse	58	22	172	3.9	Yes	D	Q95182	1EW3	(27)
Equ c 2^g^	Horse	*50*	16		3.4–3.5	No		P81216 P81217		(28)
Fel d 4	Cat	47	20	171	4.6	Putative		Q5VFH6		(29)
Fel d 7	Cat	*Up to 47*	18	154	4.9	No	D	E5D2Z5		(30, 31)
Mes a 1	Golden hamster		20.5, 24, 30	157		Both		Q9QXU1		(32)
Mus m 1	Mouse	*66*	18–21	162	4.6–5.3	No	M/D	P02762	1MUP	(33, 34)
Ory c 1^g^	Rabbit		17–18			Yes				(35)
Ory c 4	Rabbit	*46*	24	172				U6C8D6		(36)
Phod s 1	Siberian hamster		18, 21, 23	151				S5ZYD3		(37)
Rat n 1	Rat	*66*	17–21	162	4.2–5.5	Yes	M/T	P02761	2A2U	(33, 38)

a *Modified from (14) with permission from Taylor & Francis Group LLC—Books*.

b *Approximate average percentages of source–exposed symptomatic and source–sensitized subject groups based on information at www.allergome.com. Upon insufficient information at the database, sensitization rates (in italics) are from key references*.

c *Molecular mass*.

d *M, monomer; D, dimer; T, tetramer*.

e *UniProtKB, https://www.uniprot.org*.

f *Protein Data Bank, https://www.rcsb.org*.

g *Only N-terminus known*.

Lipocalin proteins have three large structurally conserved regions (SCR1-3) in their fold (17, 39). Most lipocalins contain SCR1 and SCR3. SCR2 is present in more than 60% of lipocalins. Kernel lipocalins have all three SCRs while outlier lipocalins have one or two of them. SCRs contain characteristic sequence motifs.

The three-dimensional structure of lipocalins is similar regardless of the low sequential identity (17, 39). It comprises a central β-barrel which is composed of eight antiparallel β-strands. Importantly, the β-barrel encloses an internal hydrophobic ligand-binding site, with a loop scaffold at its entrance (17, 19, 39). At the N-terminus, there is a 3_10_ helix and at the C-terminus an α-helix of variable length. The three-dimensional structures of several lipocalin allergens have been resolved (Table 1). Lipocalins may form covalent and non-covalent complexes with soluble macromolecules (17, 19).

From the standpoint of innate immunity, one of the properties of lipocalins potentially important for their allergenicity is their capacity to carry diverse molecules in their ligand-binding site, the cup-shaped cavity (19, 39). The structure of the cavity varies allowing different lipocalin molecules to harbor ligands of various sizes and shapes (19, 39). Therefore, a repertoire of small, principally hydrophobic molecules, such as odorants, pheromones, steroids, vitamins, and lipids are bound by mammalian lipocalins (19, 39). Experimentally, individual lipocalin proteins are often able to bind several diverse ligands, and therefore the physiological significance of a ligand can remain unclear (19, 40). Obviously, some lipocalins exhibit higher selectivity for ligand binding than others, for example, retinol-binding protein more than lipocalin-1, a multifunctional protein (40, 41).

Another characteristic feature of lipocalin proteins is their capacity to bind to specific cell-surface receptors (39, 42). In humans, three groups of lipocalin receptors can be recognized (42, 43). The group taking up the lipocalin-ligand complex in an endocytic manner includes megalin (44), the lipocalin 1-interacting membrane receptor (LIMR) (41, 45), the lipocalin-2 receptor NGalR/24p3R (46) and basigin (47). In the second group is STRA6, a receptor for retinol-binding protein, that is part of the transfer mechanism of retinol to cells and does not comprise endocytosis (48). The third group contains CD45 which is a receptor for glycodelin (placental/pregnancy protein 14), involved in reproduction and immune modulation (39, 49). Glycodelin, a lipocalin lacking a pronounced central cavity (50), exerts its function via CD45 by attenuating the signaling of the antigen receptor of T lymphocytes, the T cell receptor (TCR) (51–53). Mouse major urinary proteins (MUPs), in addition to being pheromone carriers, act as pheromones themselves by binding to their neuronal receptors (54–56). Interestingly, two homologous lipocalins with the mouse allergen Mus m 1/MUP6, the rat MUP13 and the cat allergen Fel d 4, were found to function as kairomones in mice (57).

Lipocalins are involved in multiple other functions, such as olfaction, gustation and prostaglandin D_2_ synthesis (19, 39, 56). Several lipocalin proteins are acute-phase proteins, thus related to inflammation and innate immunity (58, 59). Therefore, neutrophil gelatinase-associated lipocalin or alpha-1-acid glycoprotein, for example, may be used as inflammatory biomarkers in medical conditions (60, 61). Lipocalins are not usually enzymes, a feature shared by several allergens, but the exceptions are lipocalin-1 and the food allergen Bos d 5 which have non-specific endonuclease activity, and the lipocalin-type prostaglandin D synthase (19, 62).

## Lipocalin Allergens

Most mammalian lipocalin allergens are implicated in the respiratory allergy (Table 1). Milk allergen Bos d 5 (β-lactoglobulin) and the arthropodan allergens cockroach Bla g 4 (*Blattella germanica*) and Per a 4 (*Periplaneta americana*), the “kissing bug” (*Triatoma protracta*) Tria p 1 and the pigeon tick (*Argas reflexus*) Arg r 1 also belong to lipocalins.

Currently, the Allergen Nomenclature Database contains lipocalin allergens from 10 mammals, including two important indoor allergen sources, cat and dog (Table 1). Altogether there are 19 mammalian lipocalin allergens, dog, and guinea pig having the highest number of them, four allergens (out of 8 and 5, respectively). Table 1 shows that lipocalin allergens sensitize efficiently, the approximate average sensitization rate being more than 50% according to different studies. Their common features also include, for example, molecular size and an acidic isoelectric point whereas glycosylation and oligomeric state differ between them.

Although the level of amino acid identity between lipocalins is in general low, lipocalin allergens can exhibit higher identities, over 50%, in several cases (14). For example, it is 67% between cat Fel d 4 and dog Can f 6. The identity between the dog allergen Can f 1 and human lipocalin-1 (LCN1; tear lipocalin/von Ebner gland protein) is 61%. Obviously, the sequential similarity between lipocalins/lipocalin allergens is reflected in their IgE cross-reactivity and can have a role in their cellular immune recognition (14, 34, 63).

Lipocalin allergens share the preserved three-dimensional structure with other lipocalins (17, 19, 39, 64, 65). One characteristic feature of the lipocalin structure is its tendency for oligomerization (arrangement in multisubunit complexes) and complexation with soluble macromolecules (39). Accordingly, lipocalin allergens are prone for forming oligomers, such as dimers and tetramers (Table 1). They are mostly transient in nature (65, 66). Although oligomerization is probably not linked to the allergenicity of lipocalins (i.e., capacity to induce type 2 immunity), it can be a property of an allergenic molecule, as it presumedly facilitates IgE cross-linking on immune cells by offering identical epitopes.

Solubility in water and stability have customarily been associated with allergenic molecules (67). Obviously, lipocalin allergens are water-soluble because they are present in various animal excretions. Lipocalins are mostly considered exceptionally stable, for example, against thermal and chemical denaturation (55). Accordingly, the common presence of lipocalin allergens in the indoor environments even without pets should not be a surprise. Recent reviews, however, illustrate well that the physicochemical characteristics of allergens in general and those of lipocalin allergens in particular are challenging to translate into understanding the allergenicity of proteins, and that includes their abundance in the source (68, 69). For example, the sensitizing capacities of dog Can f 1 and Can f 4 are roughly at the same level [Table 1 and (14)] despite their different proportions in the dog dander extract (10 vs. 0.5%, respectively) (63). However, it should be taken into account that their inhaled proportions are not known.

All things considered, the physicochemical properties of lipocalin allergens can offer some cues for explaining their allergenic capacity. Although incompletely understood, the fact that a major part of mammalian inhalant allergens concentrate in lipocalins, it is justified to suppose that there are specific features that promote their allergenicity. Whether this is intrinsic to the molecules themselves or related to external factors or substances associated with them, is still unresolved. Even if the latter were the case, there remains an issue as to how a proportion of environmental proteins is selected as allergens; it is well-known that not all proteins in an allergen source are allergenic. For example, one study found that 30–40% identity with self proteins may be optimal for the allergenic capacity of a protein (70). For the uniqueness of allergens among antigens also suggests the observation that they exist in only 2% of more than 9,300 protein families analyzed and that 10 of these families contained more than 40% of the allergens (71).

## Allergic Sensitization

In addition to the erroneous T-helper type 2 (Th2) response to allergens, type 2 immunity is involved in several beneficial roles, such as protection to helminths (72), tissue repair (73), and metabolic homeostasis (74). In type 2 immunity, CD4+ T cells and their cytokines, in particular, IL-4, IL-5, IL-9, and IL-13, are of outmost importance. Yet, other cell populations, such as myeloid cells and innate lymphoid cells (ILCs), participate in the sensitization process, immediate IgE-mediated allergic reaction, the following allergic inflammation and its long-term consequences (4, 75, 76).

In the context of allergenic capacity of lipocalins, or allergens in general, the basic question is what are the factors which determine that specific IgE is produced against them—and not to a spectrum of other proteins present in the environment. One such factor is obviously IL-4. As this cytokine plays a crucial role in the Th2 differentiation, it needs to be present when professional antigen-presenting cells (APCs), such as dendritic cells (DCs), macrophages or B cells, present processed allergen peptides in association with the major histocompatibility class II (MHCII) molecules to naive CD4+ T cells (77, 78). In other words, IL-4 has to be available at the early phase of the APC-T cell contact. As the potent APC, dendritic cell, has not been discovered to synthesize IL-4 (75, 79) and specific positive regulators for Th2 differentiation in DCs have not been found (79, 80), recognizing the missing pieces that promote the IL-4-predisposed microenvironment in the lymph node with allergenic proteins most probably also provide insights for the allergenic capacity of lipocalin allergens.

Although IL-4 is reported to be dispensable for Th2 differentiation in some (mostly parasite) *in vivo* models (81–84) and the stimuli required for Th2 differentiation are discussed (79, 80, 85–87), the role of IL-4 is well-recognized: it is a central factor for the canonical Th2 differentiation *in vitro* and *in vivo* (77, 78, 88, 89). One possible source for early IL-4 upon antigen presentation (by DCs) are CD4+ T cells, as the TCR signal strength (a result of TCR affinity, peptide-MHCII density plus costimulation) affects Th1/Th2 differentiation, weak TCR signaling promoting their IL-4 production and the development into Th2 cells, observed in several experimental settings (79, 83, 89–94). Although this concept has aroused criticism and can be context-specific (95–97), it has even been suggested that the TCR signal strength may in some situations supersede other more typical factors, such as cytokine and costimulatory signals, associated with Th differentiation (89, 98). Therefore, further clarifying investigation is needed in this field. Another source for IL-4 could potentially be natural killer T (NKT) cells which are able to recognize lipids in association with the CD1a molecules, though their significance for allergic sensitization is ambiguous (99) and not reported to be linked to lipocalin allergens. On the other hand, non-professional APCs with capacity for IL-4 production and antigen presentation could play a role in the initial sensitization phase. As an example, one such cell population are basophil leukocytes; they are known to be able to produce IL-4, to express costimulatory molecules and to present antigen in association with MHCII *in vitro* and *in vivo*, and thereby to induce naive CD4+ T cells to differentiate into Th2 cells (100–102).

The low surface expression of MHCII on basophils compared to professional APCs has aroused concern on their factual capacity for antigen presentation (103, 104). However, this feature, low-level antigen presentation, may in fact be favorable for inducing Th2 development (83, 105, 106). That was already suggested earlier by murine studies focusing on peptide-MHCII ligand density on APCs (107–109). As to the reasons for the low-level MHCII expression on basophils, it can probably be explained by the finding that basophils get their peptide-MHCII complexes from DCs through the mechanism called trogocytosis (106, 110). Although further discussion on non-professional APCs is beyond the scope of this article, it can be mentioned that other cell populations, such as mast cells (111–115), eosinophils (116–119), and type 2 ILCs (ILC2) (120–124), are also reported to be capable of MHCII expression and IL-4 production and are therefore candidates for allergenic APCs. It is worth noting in this context, however, that the immune characteristics of lipocalin allergens themselves may favor the CD4+ T cell-driven Th2 development (14, 63, 125): We have discovered that the lipocalin allergen bovine Bos d 2 is poorly immunogenic in a mouse model (126, 127), several lipocalin allergens are weakly antigenic *in vitro* (128–132) and that the T cell epitopes studied in Bos d 2 and Can f 1 are recognized suboptimally by human T cells (133–135). Thus, one factor for the allergenicity of lipocalins can be that they are at the borderline of self and non-self in that proteins too close to the immunological self are non-immunogenic and therefore tolerated, the proteins evolutionary distant, for example bacterial proteins, are strongly immunogenic and therefore do not (in general) elicit a type 2 response whereas the proteins able to cross the threshold for TCR activation without being too stimulatory have the potential being allergenic (14, 63, 125). This view is compatible with the finding that 30–40% identity with self proteins may be optimal for the allergenic capacity of a protein (70), because mammalian lipocalin allergens often fulfill that requirement.

For the class switch recombination to take place for specific IgE production, B cells require T cell help. This occurs through the interaction between the surface molecules CD40 on B cells and CD40L on T-follicular helper (Tfh)/Th2 cells plus the action of the cytokines IL-4/IL-13 (136, 137). Of interest, a recent report described a rare IL-13-producing Tfh cell subset (Tfh13) which was required for inducing high-affinity IgE to allergens (138).

Even though allergic inflammation and allergic sensitization are interrelated phenomena, it is plausible that type 2 inflammation can take place independently of classical IL-4-producing Th2 cells (139–142). In the context of allergenicity of proteins, as IL-4 still plays such an obvious role in allergic sensitization, a safe approach for assessing the sensitizing capacity of a protein is to monitor for IL-4 signaling in the appropriate *in vitro/in vivo* setting with naive CD4+ T cells (and/or specific IgE production). As shown by examples below, innate immune responses associated with allergens can be variable in regard to IL-4 signaling/production.

## Innate Immunity in Shaping Allergen-Specific Sensitization to Lipocalin Allergens

### Innate Immune Response at Barrier Sites

The integrity of the body's epithelial barriers has been realized to be of fundamental importance for epithelial homeostasis upon exposure to environmental substances (143). Several external factors, such as opportunistic or pathogenic microorganisms, trauma and tissue injury, pollution and allergens, however, can deteriorate the proper function of the epithelial barrier (144). Upon such insults, a variety of pathogen-associated and/or damage-associated molecular patterns (PAMPs and DAMPs, respectively) are recognized by the cognate receptors of innate immune and other cells (pattern recognition receptors, PRR) resulting in the initiation of immune response (143–146). Here, epithelium-derived cytokines, in particular thymic stromal lymphopoietin (TSLP), IL-33, and IL-25 (alarmins), act as a front-line warning of the disturbed epithelial integrity (147). In addition to epithelial cells, these cytokines are produced by a wide range of other cell types, for example stromal cells, fibroblasts, mast cells and eosinophils, and likewise they have pleiomorphic effects on numerous cell populations (147).

TSLP, IL-33 and IL-25 are mostly associated with type 2 inflammation (147–151). They are also implicated in the differentiation of naive CD4+ T cells into Th2 phenotype, as shown by a parasitic nematode model in which the terminal differentiation of effector Th2 cells failed when all three cytokine signals were lost (152). As to TSLP alone, its capacity to induce Th2 immune deviation depends on TCR engagement and IL-4 (153–156). In addition, TSLP can strengthen ongoing Th2 effector and memory responses (156, 157). One study found it dispensable in generating IL-4-dependent humoral and cellular immunity to house dust mite (HDM) and peanut allergen extracts (158). Of interest, TSLP was observed to induce a unique IL-13-single positive Th2 population originating from the IL-4-negative precursors of naive murine CD4+ T cells in a Th2-promoting culture setting containing IL-4 (142). In models of *Trichuris muris* infection, TSLP promotes the type 2 immune response by preventing IL-12 production by DCs (159, 160). It was not required for the protective outcome in infections with two other helminth strains (160).

IL-33 is released by several cell types, in particular epithelial and endothelial cells, following cell injury or death (147, 150, 151). While IL-33 was originally observed to be involved in type 2 inflammation, it is also reported to play a role, for example, in protecting tissues (161, 162), even in allergen-induced inflammation (163, 164), and in the antitumor immunity with IFN-γ production by tumor-infiltrating CD8+ T cells (165). It is a pleiotropic cytokine mostly affecting a variety of allergic inflammation-related cell types, including memory Th2 cells (147, 150). While IL-33 was primarily seen to be implicated in the overall adaptive immune priming of naive CD4+ T cells in a mouse model, especially with HDM allergen extract (158), another study found that naive murine CD4+ T cells which were incubated with IL-33-activated DCs (with or without antigen) produced IL-5 and IL-13 but not IL-4 (139). A respective result was obtained in another murine study (140). One study showed a weak IL-4 production in the bronchoalveolar lavage fluid (BALF) in response to 4-day IL-33 airway challenge (166). Two other murine models with papain or *Alternaria alternata* administered through airways also observed the IL-33-dependent release of IL-13 (and IL-5) in lungs but the release of IL-4 was not reported (167, 168). In a more recent murine model of intranasal sensitization with the protease allergen papain, the IL-33-mediated activation of ILC2s was found critical in that IL-33 induced ILC2s to produce IL-13 (84). Analyzed 6 days later after administration of OVA plus papain intranasally, IL-13 was found to be involved in the differentiation of classical IL-4-producing Th2 cells in mediastinal lymph nodes in the TCR-transgenic model exploiting OVA-specific OT-II T cells. One more study found that intranasal sensitization with OVA together with IL-33 resulted in the production of IL-4 by Tfh and Th2 cells from mediastinal lymph nodes upon *in vitro* stimulation with OVA; Tfh cells did not produce IL-5 and they produced less IL-13 than Th2 cells (137). Finally, recent human studies reported that the IL-33-enhanced production of IL-4 and IL-5 by terminally differentiated Th2A cells was dependent on TCR recognition (169, 170).

As some allergen sources contain enzymatic components, it is of interest that full-length IL-33 is cleaved into shorter mature forms with increased activity by a range of allergen preparations in experimental settings, resulting in the production of IL-5, IL-13, and IL-6 (171). In another recent work, mite group 13 allergens, fatty acid-binding proteins, were reported to be recognized by the acute-phase protein serum amyloid A1 (SAA1) (172). The interaction of SAA1 with the allergens resulted in the formyl peptide receptor (FPR) 2-dependent epithelial release of IL-33 and allergic inflammation. IL-4 synthesis was not reported. In a gastrointestinal helminth model, TCR-mediated recognition allowed Th2 cells to express epidermal growth factor receptor (EGFR) and amphiregulin which in turn allowed Th2 cells to secrete IL-13 upon exposure to IL-33 (173). The effect was IL-13-specific, as *il4* and *il5* mRNA expression in mice lacking EGFR was unaffected at the site of infection.

As TSLP and IL-33, IL-25 is a pleiotropic cytokine, released by and affecting a number of cell types (147, 174). Important recently described sources of IL-25 are tuft cells in the intestine and lung of mouse (175) and solitary chemosensory cells in the human upper airways (176). IL-25 is a strong amplifier of Th2 immunity its targets being, for example, CD4+ T cells and ILC2s (147). Infusing mice with IL-25 induces many of the features of type 2 inflammation, such as the production of IL-4, IL-5, IL-13, and the increase of serum IgE, eosinophilia and mucus production (177). A murine model of persistent allergic airway disease showed that the upregulation of IL-25 induced the accumulation of a granulocyte population producing IL-4 and IL-13 in the lung (178). IL-25 is not only able to potentiate the function of effector Th2 cells but it can induce the Th2 differentiation of naive CD4+ T cells in an IL-4 and signal transducer and activator of transcription 6 (STAT6)-dependent fashion upon TCR engagement *in vitro* in a mouse model (179). However, another murine study reported that IL-25 signaling is not required for generating Th2 immunity to HDM or peanut extracts *in vivo* (158). In a murine model of HDM asthma, IL-25 directly stimulated Th2/Th9 cells to produce IL-13 and IL-9, and the role of CD11c+ DCs was important in that their stimulation with IL-25 promoted the local accumulation of the T cells (180).

Other epithelial cytokines often associated with the development of type 2 inflammation include granulocyte-macrophage colony-stimulating factor (GM-CSF) and IL-1. In a model of intranasal sensitization with cockroach allergen extract, GM-CSF was required for allergic inflammation and sensitization (181). The model was completely dependent on CD4+ T cells. In the model, blocking GM-CSF reduced the production of IL-4 and IL-13 by lung lymph node-draining cells while its administration boosted the production of IL-5 and IL-13 by lung CD4+ T cells upon TCR engagement (181). A model of intranasal sensitization with the dust mite *Blomia tropicalis* extract showed that neutralizing GM-CSF attenuated Th2 priming whereas the administration of the cytokine enhanced the proliferation and Th2 immune deviation of CD4+ T cells (182). Another murine model, here with HDM extract and/or ovalbumin (OVA), pointed out that CM-CSF signaling was implicated in Th2 polarization in some experimental settings (183). Other studies with HDM extracts also suggest that the blockade of GM-CSF signaling diminishes allergic inflammation (184–186). It appears that more research for clarifying the significance of GM-CSF for Th2 differentiation is needed (79, 89, 145).

IL-1, like the other cytokines discussed here, exerts multiple functions in the innate and adaptive immunity, and is also involved in the immunopathological conditions, such as autoimmunity (187). In a HDM asthma model, IL-1a was released from epithelial cells in a Toll-like receptor (TLR)4-mediated manner (185). In one of the subsequent experiments, exploiting OVA-specific TCR-transgenic OT-II cells, IL-1a was found to promote the production of IL-4, IL-5, IL-17 and IFN-γ by the T cells upon stimulation with OVA. In another *in vivo* study with OT-II cells, IL-1 enhanced the expansion of Th1, Th2, and Th17 cells (188). One more study showed that IL-1a was dispensable for allergic responses in a sensitization model with cockroach allergen extract (181). It seems that the role of IL-1 in type 2 immunity is limited (145, 187, 189, 190). Of interest, however, an asthma model with HDM extract showed that Th17 cells, induced by epicutaneous immunization, can switch their phenotype to Th2 upon intratracheal challenge (94). Therefore, an initial allergen-specific immune response with Th17 polarization may result in the mixed Th17-Th2 response to allergen in the later phase, and to severe asthma.

A cytokine not categorized as epithelium-derived or an alarmin, IL-12, may also be implicated in promoting type 2 immunity in that its absence can favor the Th2 development. In a study with human cells, it was found that TSLP-treated CD11c+ DCs did not produce IL-12 (in contrast to CD40L and lipopolysaccharide (LPS)-activated ones), and that they were able to induce naive CD4+ T cells to produce large amounts of IL-13, IL-5, and tumor necrosis factor (TNF) plus a moderate amount of IL-4 upon TCR engagement (191). Another study extended these observations and found that OX40L on TSLP-activated DCs in the absence of IL-12 triggered the Th2 polarization which was critically stabilized and enhanced by IL-4 (192). Later, it has been found that TSLP-activated DCs were able to induce human Tfh2-resembling cells with ability to promote IgE secretion from memory B cells that depended on IL-4Rβ (155). Recently, a model of atopic dermatitis (AD) revealed an interesting connection between innate and adaptive immunities (193). In this TCR-transgenic model, mechanical skin injury drove the IL-33-dependent production of IL-13 by mast cells which suppressed IL-12 production by skin-derived DCs. This was supposed to favor the induction of Th2 responses in AD although IL-4 production was not investigated. It seems that one of the mechanisms through which type 2 conventional DCs support Th2 responses is the reduced expression of IL-12 (79).

As exemplified by studies reviewed above, innate immunity-focused mechanisms accounting for the allergenic capacity of lipocalin allergens are practically missing. Consequently, it is of interest that a novel mechanism for their allergenicity, operative through FPR3 in DCs, was recently proposed (194). Two lipocalin allergens, dog Can f 1 and cat Fel d 4, were found to hinder IL-12 production by human monocyte-derived DCs which was not the case with human non-allergenic counterparts of the allergens, lipocalin-1 and major urinary protein (pseudogene). Moreover, naive allogeneic CD4+ T cells cocultured with DCs together with Can f 1 or Fel d 4 produced more IL-4 and IL-13 than the cells cocultured with FPR3-silenced DCs plus the allergens. Thus, downregulation of IL-12 production by DCs through the activation of FPR3 can be a mechanism for the Th2-polarizing capacity of lipocalin allergens (a total of five allergens were examined). As the group's previous work also showed that the expression of CD40, CD80, and CD83 (but not MHC II and CD86) were significantly lower in DCs treated with Can f 1 than lipocalin-1, Can f 1, an exogenous protein, appears to be (surprisingly) less stimulatory for DCs than the endogenous human protein (195). It is conceivable that this can result in the less efficient TCR engagement and therefore further Th2 polarization, as discussed above. However, an earlier study comparing human T cell responses to Can f 1 and lipocalin-1 found that although Can f 1 was slightly more efficient in inducing specific T cell lines than lipocalin-1, the proliferative and cytokine responses of the Can f 1-specific T cell lines were largely similar as those of lipocalin-1-specific T cell lines (132).

Other cytokines sometimes mentioned, for example the proinflammatory type I interferons (INF-I) and TNF, require further research for evaluating their role in the induction of type 2 immunity to allergens (80, 145, 189, 196). Yet, TNF appears to be involved in LPS-dependent allergic sensitization (189). In the asthma model, OVA together with TLR ligands (LPS/flagellin) or house dust extract (HDE) as adjuvants induced a similar accumulation of TNF, IL-1a, IL-1b, and GM-CSF in BALF pointing to the possible role of LPS in the effect with HDE. In a protease-mediated asthma model, TNF was dispensable (189). TNF also appears to be involved in impairing the integrity of bronchial epithelial barrier (197).

Upon activation of the airway (and other) cells, one of the innate (and adaptive) immune responses can be the release of exosomes, a subtype of extracellular vesicles (198, 199). They act as mediators of intercellular communication containing diverse types of cargo depending on the cell type. In asthma, exosomes are mainly involved in the pathogenesis of the disease promoting inflammation. In a recent study, exosomes released from airway epithelial cells primarily promoted general immune responsiveness and type 2 inflammation in a mouse model of sensitization with HDM extract (200). Of interest, human B cell-derived exosomes loaded with allergen peptides were shown to induce specific T cell response and the exosomes from monocyte-derived DCs were reported to be able to carry the Fel d 1 allergen (201, 202). Although the significance of these observations requires further clarification, it should be noted that the selective sorting of microRNAs via exosomes may be one mechanism among others to regulate type 2 responses (199, 203).

As delineated above, the epithelium-associated innate immune responses are fundamentally implicated with allergic inflammation, but allergic inflammation *per se*, or cytokines associated with it, do not directly switch on and sustain the canonical allergen-specific sensitization manifested by IL-4-producing CD4+ T cells. In other words, allergen-specific sensitization essentially depends on CD4+ T cells which recognize allergen peptides by their TCRs, which obtain IL-4 signal for their differentiation, and which produce IL-4 (89, 145). As exemplified by the studies above, the CD4+ T cells themselves are largely the source for IL-4 when they become activated through TCR. Accordingly, innate immune response at barrier sites is not able to account for the specificity (or target) of sensitization, whether to lipocalin or other allergens. Moreover, allergen preparations used in several experimental settings are extracts, not pure allergen molecules, and this obviously obscures the role of an allergenic protein in the process. In summary, it seems for the time being that epithelial innate immune responses are not able to explain the allergenicity of specific proteins, including lipocalins. Therefore, as cited above, it is of interest that FPR3 in DCs and the downregulation of IL-12 is reported to promote the allergenicity of lipocalin allergens (194). Here, it should be noted though that simple blocking of Th1-inducing stimuli, such as IFN-γ or IL-12, may not be sufficient for Th2 differentiation (204, 205).

### Receptors of Innate Immunity Recognizing Lipocalin Allergens

Because a characteristic feature of lipocalin proteins is their capacity to bind to specific cell-surface receptors (39, 42), as discussed above, it is tempting to speculate about the role of this phenomenon for their allergenicity. For example, food allergen β-lactoglobulin (Bos d 5) was reported to be taken up by LIMR (206) which was not, however, confirmed in another study (207). Instead, β-lactoglobulin, as well as cat Fel d 4, were recently found to be internalized by the cell-surface heparan sulfate proteoglycan-mediated endocytosis in HeLa and CHO cells (43). Although these studies did not assess the allergenic capacity of lipocalins, obviously the active uptake of allergens through binding to cell-surface receptors can facilitate their access to the immune system. As mentioned above, Th2 polarization has been reported to be promoted by the uptake of lipocalin allergens through the FPR3-mediated mechanism in DCs (194, 195). Moreover, the endogenous lipocalin, glycodelin, favors Th2 immune deviation by binding to its cell-surface receptor CD45 and thereby attenuating TCR signaling (51–53).

The glycosylated dog allergen Can f 1 has been reported to be recognized by two distinct C-type lectin receptors (CLR), mannose receptor (MR) and dendritic cell-specific intracellular adhesion molecule (ICAM)-3-grabbing non-integrin (DC-SIGN, CD209). Results concerning these two receptors are conflicting in that the supposed outcome, Th2 polarization, was only favored by the recognition with MR (208). In this study focusing on MR, CD3+ autologous T cells from HDM-sensitized subjects were cocultured with DCs in the presence of a glycosylated (non-lipocalin) allergen, mite Der p 1, and LPS. As low-level LPS stimulation is known to promote Th2 immunity (209, 210), its role might have been important in the process. The experimental setting of the other study focusing on DC-SIGN was otherwise comparable, but it included naive CD3+ autologous T cells from a non-atopic donor and no LPS. In this case, recognition by DC-SIGN favored the opposite development, Th1 immune deviation (211). In one additional study, a dog lipocalin allergen, Can f 6, was found to enhance LPS-induced activation of TLR4 signaling assessed by the TNF production of murine bone marrow–derived macrophages, in a somewhat similar manner as the major allergen of cat, Fel d 1 which is not a lipocalin (212). A more detailed analysis on Fel d 1 showed that the effect was independent of MR activity. The model was not demonstrated, however, to be able to elicit sensitization. Yet, Fel d 1 is reported to be internalized by human and mouse APCs by a MR-mediated mechanism (213). This recognition conceded a Th2-polarized immune response to the allergen in a mouse model (213). As to the role of glycosylation for the allergenicity of lipocalins, it should be noted that only part of them are glycoproteins (Table 1).

Indeed, TLRs and CLRs together with other PRRs, such as NOD-like receptors (NLR), RIG-I-like receptors (RLR) or protease-activated receptors (PAR), are implicated in the allergic sensitization and/or inflammation (214–218). As exemplified above, their effects on allergenic processes appear to be variable, depending on various factors such as the dosage of ligands, in that they can either enhance or prevent allergenicity (214, 215, 217, 218).

In this context, it is important to pay attention to the sensitization-associated ligands that PRRs recognize because these ligands may constitute an integral part of an allergenic molecule, such as a glycan moiety, or they are just present (more or less) regularly, or just occasionally, with the allergen. Of note, simple non-integral presence of PAMPs/DAMPs with an allergen preferably points to an (extrinsic) adjuvant effect rather than to an (intrinsic) allergenicity of the protein. Thus, it is important to consider whether a given protein can function as an allergen without extrinsic codelivered immunomodulatory components, possible environmental contaminants, or other substances. A recent study on peanut allergens illustrates well the complexity of this issue: Even though peanut extract was able by signaling through TLR2 to strongly induce the expression of *ALDH1A2*, the gene encoding retinaldehyde dehydrogenase 2 (RALDH2), in human myeloid (m) DCs and monocytes, and to increase the activity of the enzyme 7-fold in mDCs, the *ALDH1A2*-inducing activity, related to Th2 differentiation, could not be attributed to the pure peanut allergens or other peanut proteins examined (219).

As to LPS, known to be important for Th2 polarization at a low concentration (209, 210, 220), is an adjuvant (see more on adjuvants, below). Notably, the majority of studies for the time being has employed allergen extracts which are known to contain LPS and other contaminating material (220–222). In case of mite Der p 2, a non-lipocalin allergen, the proposed [and debated (223, 224)] allergenic mechanism is though interesting because the allergen exhibits structural homology with MD-2, a LPS-binding component of the TLR4 signaling complex (221). Thereby, it was found to facilitate signaling through direct interactions with the complex (221). For lipocalin (or other) allergens, this type of a “self adjuvanticity,” or allergenicity, has not been described.

Taken together, there are relatively little data concerning the recognition of lipocalin allergens by the characteristic lipocalin receptors or other receptors of the innate immune system in the context of specific allergic sensitization. Therefore, the receptor-ligand systems studied so far incompletely reveal the rationale behind the allergenicity of lipocalins. As mentioned above, specific costimulatory or cell-surface ligands selectively inducing Th2 differentiation have not been identified for example in DCs (79), with a possible exception of FPR3 for lipocalin allergens (194). Obviously, the specific recognition of lipocalin allergens by the receptors of innate immune system in explaining their allergenic capacity requires further research.

### Adjuvants Associated With Lipocalin Allergens

Adjuvants, substances appearing naturally or codelivered with immunogens (vaccines), can strongly influence the quality and strength of a specific immune response. Part of allergen-associated adjuvants can derive from environmental pollution, such as diesel exhaust particles (225). In the allergen immunotherapy, adjuvanticity is exploited in that a few adjuvants, most notably aluminum hydroxide (alum), are used to promote a favorable outcome, tolerance to the allergen (226). A little surprisingly in the context of allergy, alum has been found to boost Th2 immunity, as do some other inert particulate materials causing sterile inflammation, such as silica or uric acid crystals or micrometer-sized metallic particles, apparently as a consequence of IL-4 synthesis and/or absence of IL-12/IFN-γ (227–230).

Helminth parasites typically employ multiple mechanisms to suppress host defense and to establish a chronic infection with type 2 immunity (231, 232). One way the helminths can achieve this outcome are various immunomodulatory products that they release upon infection. For example, *Schistosoma mansoni* egg-derived omega-1 induces the Th2 polarization of human allogeneic naive CD4+ T cells in cultures with monocyte-derived DCs upon TCR engagement in a MR (but not DC-SIGN)-dependent manner (233). An *in vivo* mouse model showed comparable results. The authors discussed that due to the impairment of DC function by omega-1, T cells may be primed in the absence of IL-12 with low-level antigen presentation, as this combination would favor the Th2 response (233). In another murine *S. mansoni* infection model, T cell-derived IL-4/IL-13 were found essential in providing protection against the fatal course of the disease. Here, basophils were dispensable as a source of IL-4/IL-13 for Th2 polarization (234).

Plants also contain substances that can influence the quality of an immune response (235). One example is the immunity to birch pollen. *Betula alba* L. aqueous pollen extracts (APE) were found to contain E_1_-phytoprostanes (PPE_1_) which inhibited IL-12 production by DCs. Culture of allogeneic naive T cells together with LPS-activated DCs plus APE resulted in the Th2 polarization IL-4-dependently (236). Accordingly with Bet v 1 from *B. verrucosa*, the aqueous birch pollen extract but not the allergen itself was able to induce Th2 polarization, for example IL-4-producing inguinal lymphocytes in an *in vivo* mouse model after immunizations (237). As the production of Th1-related cytokines was not observed in these experiments, the Bet v 1 allergen may in fact be immunologically inert (of low/mediocre immunogenicity), similarly as are several lipocalin allergens examined (127, 238). It is conceivable that such a property can be a feature of allergens, allowing adjuvants or TCR signal strength more easily to modify the response.

Recently, the allergenicity of Bet v 1 was reported to be associated with its capacity to function as a carrier of the adjuvant PPE_1_ (239), which is of interest, as lipocalins characteristically can bind various hydrophobic ligands (19, 39). Ligand transport has also been thought to be decisive for the allergenic capacity of lipocalins (240, 241) but the issue has remained unresolved (223). In this context, however, it is reasonable to consider adjuvants which could be codelivered with lipocalin allergens. As already discussed, one of them is LPS (endotoxin) which is known to be emitted by pets (242). Being able to promote experimentally Th2 polarization at a low concentration (209), the low levels of LPS in the environment are also associated with atopic disorders in children (243, 244). LPS has been suggested to be involved in the allergenicity of dog Can f 6 by a mechanism not completely clear (212). A potential adjuvant with lipocalin allergens is nucleic acid which can be anticipated to be carried with animal-derived dust containing dead cells. Here, a study found that the TCR stimulation of naive murine CD4+ T cells *in vitro* together with various nucleic acids, including self DNA/dead cells, induced the Th2 differentiation of the cells in the IL-4 signal-dependent manner with the downregulation of T-bet and IFN-γ expression (205). In a mouse model of *M. tuberculosis* infection together with silica exposure, cell death and self-DNA release were found to prime for type 2 immunity which appeared to be further exacerbated by both self and *M. tuberculosis* DNA (245). Moreover, rhinovirus infection in an experimental human setting induced the release of host DNA and was associated with type 2 immune response with the exacerbation of asthma symptom severity (246). The results were recapitulated in a model of HDM-sensitized mice (246). Thus, as suggested previously, DNA-mediated costimulation could be a trigger for early IL-4 (247), especially upon exposure to mammalian allergens. Among other type 2 response-promoting factors released from stressed or dying cells upon exposure to allergen extracts (though not known for lipocalins) are, for example, uric acid and Charcot-Leyden crystals, trefoil factors and extracellular ATP (248, 249).

## Discussion

Because such a large proportion of mammalian inhalant allergens are lipocalins, it is attractive to concur with the idea that lipocalin allergens possess specific properties which account for their allergenicity. As reviewed here, these properties still largely remain hidden, although there are cues to trace and clarify. Nevertheless, it appears presumable for the time being that the allergenicity of lipocalin allergens hardly depends on only one factor, as is the case with type 2 immunity in general: No single switch is recognized for Th2 immunity. This view is also compatible with the characteristics of innate immunity at epithelial barriers which is obviously linked with allergic inflammation. Of note, innate immune response to allergens at barrier sites is not directly linked to the fundamental features of type 2 immunity, the specificity of sensitization (depending on the recognition of an allergenic protein) and IL-4 signaling/synthesis. Therefore, soon after the initial barrier response, the components of immunity able to target and sustain the Th2-primed response are necessary. These components are CD4+ T cells and APCs, probably DCs. The CD4+ T cells select antigens as allergens and, importantly, are mostly the principal source for IL-4 upon TCR-mediated recognition, as suggested by the studies reviewed here. DCs on their part play an important role in modifying type 2 immunity through their surface molecules, including PRRs, and cytokines, or lack of them (IL-12), the expression of which can be influenced by various factors, such as adjuvants. Other scenarios with different flavors are naturally possible, especially in *in vivo* immunity to parasites. Allergens other than lipocalins, for example those with enzymatic activity, can exploit additional mechanisms. Taken together, the allergenicity of lipocalin allergens appears to be based on the collaboration between innate and adaptive immunity deduced from their overall natural, physical, and immune characteristics.

## Author Contributions

The author confirms being the sole contributor of this work and has approved it for publication.

## Funding

This work was supported by Itä-Suomen yliopisto/University of Eastern Finland.

## Conflict of Interest

The author declares that the research was conducted in the absence of any commercial or financial relationships that could be construed as a potential conflict of interest.

## Publisher's Note

All claims expressed in this article are solely those of the authors and do not necessarily represent those of their affiliated organizations, or those of the publisher, the editors and the reviewers. Any product that may be evaluated in this article, or claim that may be made by its manufacturer, is not guaranteed or endorsed by the publisher.
